# Isorhamnetin Alleviates Steatosis and Fibrosis in Mice with Nonalcoholic Steatohepatitis

**DOI:** 10.1038/s41598-019-52736-y

**Published:** 2019-11-07

**Authors:** Munkhzul Ganbold, Yohei Owada, Yusuke Ozawa, Yasuhiro Shimamoto, Farhana Ferdousi, Kenichi Tominaga, Yun-Wen Zheng, Nobuhiro Ohkohchi, Hiroko Isoda

**Affiliations:** 1 School of Integrative and Global Majors (SIGMA), University of Tsukuba, Tsukuba, Ibaraki 305-8572 Japan; 2 Department of Gastrointestinal and Hepato-Biliary-Pancreatic Surgery, Faculty of Medicine, University of Tsukuba, Tsukuba, Ibaraki 305-8575 Japan; 3 Interdisciplinary Research Center for Catalytic Chemistry, National Institute of Advanced Industrial Science and Technology (AIST), Tsukuba, Ibaraki 305-8565 Japan; 4 Alliance for Research on the Mediterranean and North Africa (ARENA), University of Tsukuba, Tsukuba, Ibaraki 305-8577 Japan; 5 Faculty of Life and Environment Science, University of Tsukuba, Tsukuba, 305-8572 Japan

**Keywords:** Drug discovery, Liver fibrosis, Non-alcoholic steatohepatitis

## Abstract

Nonalcoholic steatohepatitis (NASH) is the most severe and progressive form of nonalcoholic fatty liver disease (NAFLD), which can lead to life-threatening conditions, however, there is still no approved drug for the treatment of NASH. In this study we used human-like NASH mouse model and treated orally with isorhamnetin at a dose of 50 mg/kg to analyze the effect of isorhamnetin on the progression of NASH. NASH-induced mice represented severe steatosis with inflammation, and fibrosis in liver accompanied with high level of liver injury markers in serum. Isorhamnetin treatment reduced intrahepatic lipid accumulation and TG content by inhibiting *de novo* lipogenic pathway in NASH-induced mice. Consistent with this, isorhamnetin-treated NASH mice showed improved liver injury markers, reduced collagen deposition as well as decreased gene expression of fibrogenic markers. Taken together, here we showed for the first time that synthesized isorhamnetin alleviates pathologic features of NASH and thus can potentially contribute to NASH drug development.

## Introduction

Nonalcoholic fatty liver disease (NAFLD) is one of the leading causes of chronic liver disease in the worldwide, with its global prevalence of 25% among adult population^1^. NASH is characterized by hepatic lipid accumulation exceeding 5% of liver weight in the absence of heavy alcohol consumption associated with the histological features of hepatocyte damage, lobular inflammation, and fibrosis. The most commonly used hypothesis on its etiology is called “Two-hit theory” in which the “first hit” usually arising from metabolic complications of obesity, insulin resistance, diabetes mellitus, and metabolic syndrome. Oxidative stress, immune system, inflammation, gut and adipose tissue-derived factors, and genetic background, grouped as the “Second hit”, are shown to play an important role in the progression of NASH from NAFLD by inducing liver injury and fibrosis. Instead of a single risk factor in natural course of NASH, simultaneous persistence of more than one metabolic disorder may play pivotal role by causing subsequent pathologic features. However, the relevant signaling pathways and molecular mechanisms responsible for its progression from NAFLD to NASH remain unexplained. This gap in our knowledge considerably limits drug development against NASH. Other than lifestyle modification through diet and exercise, there are currently no other approved treatments for NASH^2,3^.

It is worth to mention that a suitable animal model is crucial in the research progress of drug discovery. Several diet- and drug-induced mouse models have been used for their morphologic and histopathologic similarities to human NASH. Methionine and choline deficient (MCD) diet-induced NASH mice model is a commonly used aggressive NASH model, however, this model seems to develop neither peripheral insulin resistance nor obesity^4,5^. Similarly, HFD-induced NAFLD rodent model could not develop hepatic fibrosis, the most important histological predictor for NASH progression in human. Nonetheless, neither MCD diet-induced NASH mice model nor HFD-induced NAFLD rodent model could represent full NASH hallmarks if the real pathologic context of human NASH needs to be considered. Kubota *et al*. and Tsuchida *et al*. proposed a combination of diet and chemical inducers for murine NASH models, however, it requires 8 and 12 weeks until NASH resembling features occur^6,7^. Interestingly, Owada *et al*. used triple combination of diet and chemical inducers to develop human-like NASH in murine within 4 weeks^8^. Thus, in this study, we used the NASH model developed by Owada *et al*. which represents all pathologic features present in human NASH^8^.

Natural flavonoids have been shown to have bioactive effects against metabolic diseases^9–11^. Isorhamnetin is a natural flavonoid found in several plants and plant-derived foods as well as an immediate metabolite of quercetin. It has been shown to inhibit proliferation of breast and lung cancer cells^12,13^, protect HepG2 cells against oxidative stress^14^, attenuate inflammatory bowel disease^15^, and repress adipogenesis in 3T3-L1 cells^16^. Research studies including investigations in our laboratory showed its antioxidant, anti-obesity, and antifibrotic effects in rodents treated with isorhamnetin or plant extracts rich in isorhamnetin^17–19^. Its aglycone parent – quercetin was already demonstrated to possess anti-fibrotic and hepatoprotective activity^20,21^, however, reports on bioavailability of quercetin showed that a majority of the absorbed latter is found in its methylated form – isorhamnetin which is maintained in plasma longer than quercetin^22,23^. It strongly implies a potential role of isorhamnetin as a mediator of the beneficial effect of quercetin. Structurally, previous comparative analysis revealed that aglycone flavonoids exert more biological activity compared to their glycones^24^.

Those collected evidence led us to hypothesize that isorhamnetin may have efficient biological activity against hallmark features of NASH. To this end, we induced NASH in mice and treated them with isorhamnetin by oral gavage and evaluated its biological efficacy in the NASH model.

## Results

### Isorhamnetin reduced liver weight without affecting body weight in NASH-induced mice

**Figure 1:**
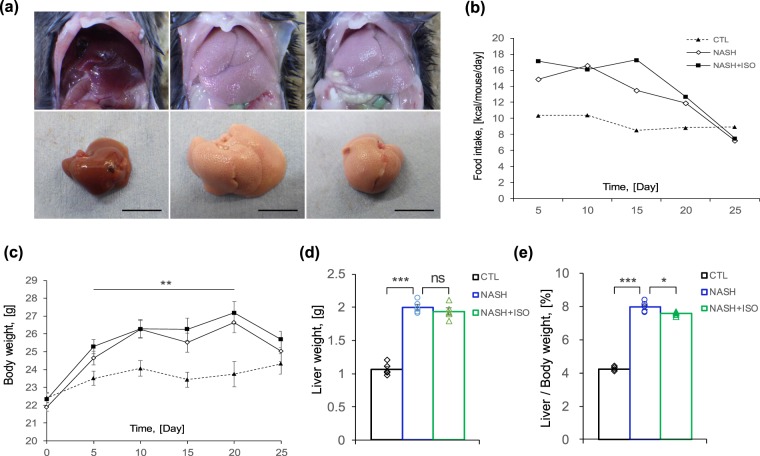
Biometrics of NASH-induced mice treated with isorhamnetin. (**a**) Representative liver aspect of experimental groups: control (CTL), NASH-induced (NASH), and NASH-induced with isorhamnetin treatment (NASH + ISO) group (scale bar = 1 cm). (**b**) Average food intake in calories of experimental groups presented as kcal per mouse per day (n = 8/group). (**c**) Body weight change (n = 8/group). (**d**) Effect of isorhamnetin on liver weight (n = 5/group, NASH vs. NASH + ISO: p = 0.389) and (**e**) liver on body weight ratio (n = 5/group, NASH vs. NASH + ISO: p = 0.025). Data are shown as mean ± SEM with significance *p < 0.05, **p < 0.01, ***p < 0.001 and ns = non-significant.

Morphologically, livers from both NASH-induced (NASH) and NASH treated with isorhamnetin (NASH + ISO) groups were significantly enlarged and pale in color indicating fatty liver aspect compared to those of control (CTL) group, which were reddish-brown in color and small-sized (Fig. 1a). Compared to CTL group, the other two groups had increased food intake in calories and weight gain. However, no significant difference was observed in daily calorie intake and change in body weight between NASH and NASH + ISO groups (Fig. 1b,c) although both groups slightly lost body weight and food intake by the end of experiment which is specific to this model. Liver weight of NASH and NASH + ISO groups were indifferent (Fig. 1d). In contrast, liver on body weight ratio, which is extensively used as a clinical parameter to assess liver enlargement, was significantly reduced in NASH + ISO mice compared to NASH group (Fig. 1e), suggesting that isorhamnetin decreased liver weight before affecting body weight.

### Isorhamnetin ameliorated liver histopathology of mice with NASH

**Figure 2:**
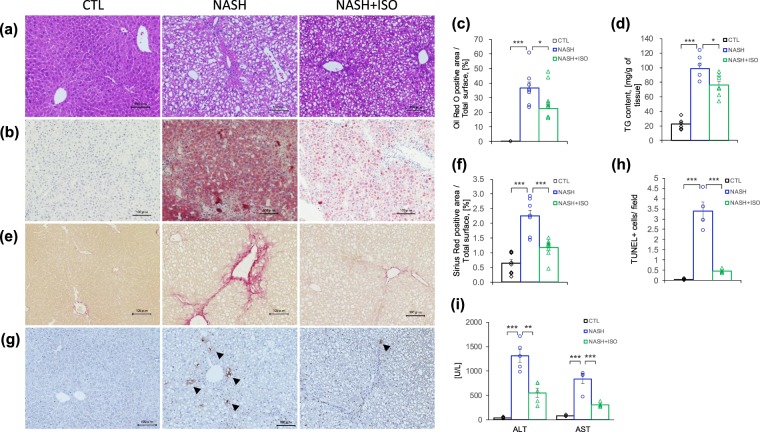
Effect of isorhamnetin on histopathology of NASH-induced mouse liver. Representative microscopic images of HE-stained (**a**), and oil red O-stained (**b**) liver sections (scale bar = 0.1 mm). (**c**) Quantification of oil red O positive area (n = 8/group, NASH vs. NASH + ISO group: p = 0.017). (**d**) Quantification of triglycerides in liver (mg/g of tissue, n = 6/group in CTL and NASH, n = 7 in NASH + ISO group, NASH vs. NASH + ISO: p = 0.029). (**e**) Representative microscopic images of Sirius red-stained liver sections (scale bar = 0.1 mm). (**f**) Quantification of Sirius red positive area of liver section (n = 8/group). (**g**) Representative microscopic images of apoptotic cells detected by TUNEL staining in liver sections (scale bar = 0.1 mm). Black arrow show TUNEL positive cells. Hematoxylin counterstaining. **(h**) Number of TUNEL positive cells per x20 field (n = 4/group). (i) Serum level of ALT and AST measured by automated biochemical analyzer (n = 5/group, ALT value of NASH vs. NASH + ISO: p = 0.0017) Data are shown as mean ± SEM with significance *p < 0.05, **p < 0.01 and ***p < 0.001.

Histopathological evaluation remains the standard method to diagnose and evaluate NASH. Therefore, liver steatosis was evaluated with Hematoxylin and Eosin (HE) and oil red O staining (Fig. 2a,b). Compared to the CTL group, NASH-induced liver exhibited severe accumulation of lipids exceeding 37% of oil red O positive area, whereas isorhamnetin treatment mitigated it until 22% (Fig. 2e). Concordant with the ameliorated steatosis, the triglycerides (TG) content in liver was also significantly reduced in NASH + ISO group compared to NASH group (76 mg/g and 99 mg/g of liver weight, respectively, p = 0.029) (Fig. 2f). This finding correlates with the reduced liver to body weight ratio observed in NASH + ISO mice.

One of the NASH features that distinguish it from benign steatosis is hepatic fibrosis characterized by excessive deposition of extracellular matrix (ECM). To address this hallmark, we evaluated collagen deposited area stained with Sirius red-stained (Figs 2c, and S1). NASH-induced liver showed greater collagen deposition surrounding portal vein than in those of CTL group. Interestingly, NASH-induced mice, when treated with isorhamnetin, had diminished collagen. The morphometric analysis further confirmed that isorhamnetin treatment reduced deposition of collagen from 2.25 to 1.17% of total surface (p < 0.001 vs. NASH) (Fig. 2g), indicating that isorhamnetin treatment efficiently improved fibrotic condition of NASH-induced liver.

Consistent damage in the liver leads to hepatocyte death by apoptosis and necrosis when the liver fails to respond adequately to this pathologic condition^25^, and increasing number of apoptotic cells contribute to exacerbating the progression of fibrosis^26^. Serum levels of alanine aminotransferase (ALT) and aspartate aminotransferase (AST), enzymes indicating liver injury state, were increased significantly in NASH-induced mice compared to CTL (p < 0.001 for both enzymes). However, NASH + ISO group had significantly decreased ALT and AST enzyme levels when compared with NASH group (p < 0.01 and p < 0.001 vs. NASH, respectively), suggesting that NASH-induced liver was less injured when treated with isorhamnetin (Fig. 2i). Moreover, highest number of apoptotic cells was found in NASH-induced mice compared to other two groups revealed by TUNEL assay (Fig. 2d,h). In addition, isorhamnetin treatment on hepatocyte and hepatic stellate cell lines did not affect the cell viability after 24 h (Fig. S2,a,b). Lipid accumulation induced by oleic and palmitic acid in HepG2 cells was not affected by isorhamnetin treatment (Fig. S2,c). These findings collectively suggest that isorhamnetin treatment alleviated histopathologic condition of NASH-induced liver by reducing steatosis, fibrosis, and number of apoptotic cells.

### Gene expression profile and related pathways following NASH-induction and changes after isorhamnetin treatment

**Figure 3:**
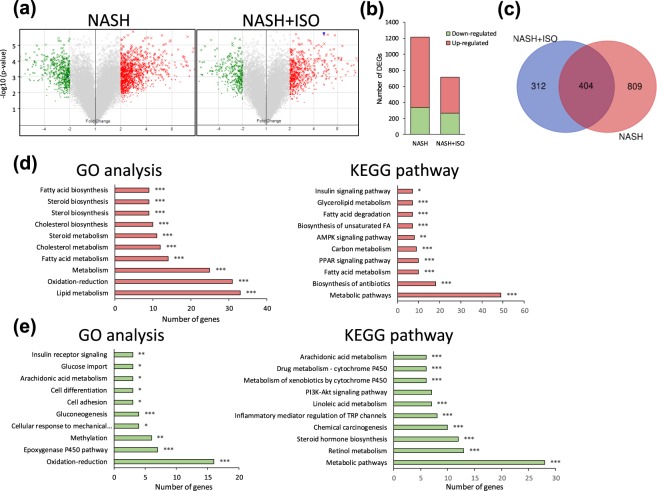
Hepatic gene expression profile in NASH and NASH + ISO group compared to CTL group. (**a**) Volcano plot of DEGs with fold-change over ± 2.0 (green: down-regulated, and red: up-regulated genes) between NASH vs. CTL, and NASH + ISO vs. CTL. **(b**) Quantitation of number of DEGs. (**c**) Venn diagram showing the overlap of DEGs between NASH vs. CTL, and NASH + ISO vs. CTL. GO process analysis and KEGG pathway for the up-regulated 257 genes (**d**) and down-regulated 147 genes (**e**) from overlapping 404 DEGs with p-value as *p < 0.05, **p < 0.01, ***p < 0.001.

To clarify the expression pattern of genes in the liver following the induction of NASH and its changes regulated by isorhamnetin treatment, we have performed a microarray gene expression analysis. Among the 45078 probe sets, the number of genes with fold change greater than ± 2.0 and p value threshold of less than 0.05 compared to CTL were considered as differentially expressed genes (DEGs) as shown in volcano plot (Fig. 3a). The number of upregulated DEGs in NASH was remarkably reduced in NASH + ISO (Fig. 3b). The 404 overlapping genes were found between NASH and NASH + ISO (Fig. 3c). In gene ontology (GO) analysis, the upregulated genes resulting from the overlapped genes were greatly associated with lipid metabolism (GO:0006629), oxidation reduction process (GO:0055114), metabolic (GO:0008152) especially fatty acid (GO:0006631) and cholesterol (GO:0008203) metabolic processes (Fig. 3d), while downregulated genes were related to oxidation-reduction (GO:0055114), epoxygenase P450 pathway (GO:0019373), and methylation process (GO:0032259) (Fig. 3e). Along with the GO process, the upregulated genes were enriched in the metabolic pathways (mmu01100), biosynthesis of antibiotics (mmu01130), fatty acid metabolism (mmu01212), and PPAR signaling pathway (mmu03320) in Kyoto Encyclopedia of Genes and Genomes (KEGG) analysis (Fig. 3d). In addition, metabolic pathways, retinol metabolism (mmu00830), and steroid hormone biosynthesis (mmu00140) pathways enriched by downregulated genes were top-ranked in KEGG pathway (Fig. 3e). These data show that isorhamnetin treatment reduced the number of altered gene expressions due to the induction of NASH.

### Changes in lipid metabolic process and inhibition of de *novo* lipogenesis

**Figure 4:**
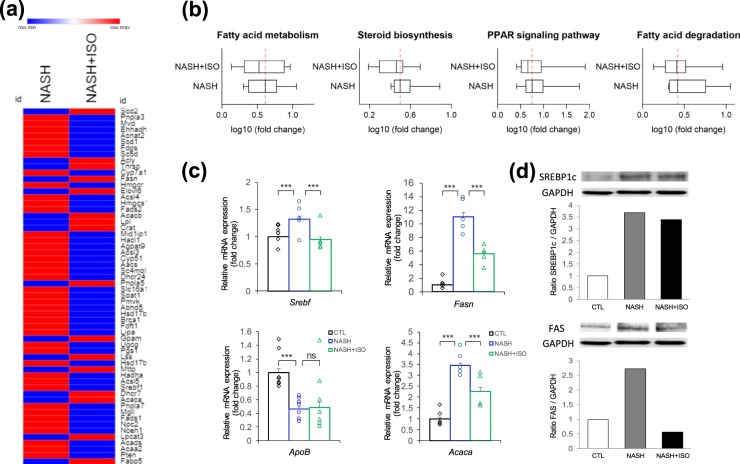
Changes in lipid metabolic process. (**a**) Heat map showing DEGs involved in lipid metabolic pathways between NASH and NASH + ISO compared to CTL group. (**b**) Box plot with median averaging genes involved in fatty acid metabolism (13 genes averaged), steroid biosynthesis (9 genes averaged), PPAR signaling pathway (10 genes averaged), and fatty acid degradation (7 genes averaged). Red dash line shows the median of NASH group (**c**) Relative mRNA expression level of de novo lipogenic genes (n = 4–6/group) by qPCR analysis. Data are shown as mean ± SEM with significance ***p < 0.001. (**d**) Immunoblot analysis for SREBP1c and Fatty acid synthase (FAS) in liver tissue and quantification of band intensities normalized to GAPDH.

The substantial numbers of genes were upregulated in lipid metabolism with the development of NASH as revealed by the GO analysis (Fig. S3). Thus, we analyzed the genes (58 genes in total) identified by heat map comparing NASH vs. CTL and NASH + ISO vs. CTL (Fig. 4a). Interestingly, the reduced level of expression for 42 genes was found in NASH + ISO compared to NASH. Next, we sought to distinguish genes by pathway axis which is involved in lipid metabolic process. As expected, the essential gene expressions in fatty acid metabolism, steroid biosynthesis, and PPAR signaling pathway were invariably decreased in NASH + ISO, while the median change of gene expression level in fatty acid degradation was not different between groups although a slight decrease in genes associated with fatty acid degradation was observed in NASH + ISO group (Fig. 4b and Supplementary Dataset). Moreover, *de novo* lipogenesis (DNL) is known to contribute nearly 30% of lipid accumulation in liver^27,28^. Thus, we evaluated the individual genes identified as the key regulators in DNL pathway such as Sterol regulatory element binding protein 1 (SREBP1c), fatty acid synthase (FAS), and acetyl-Coenzyme A carboxylase alpha (ACC1)^27,29^. We found that mRNA expression of SREBP1c, FAS, consistent with the corresponding protein level (Fig. 4d), and ACC1 was significantly upregulated (p < 0.001) in NASH-induced liver compared to CTL group, while SREBP1c-mediated DNL pathway was considerably inhibited (p < 0.001 for all genes) in NASH + ISO group when compared with NASH group (Fig. 4c). Decreased level of apolipoprotein B (*ApoB*), a rate-determining protein of lipid export in liver, is observed in NASH. The ApoB gene expression level was significantly reduced in NASH group compared to CTL but the level in NASH + ISO was similar to that of NASH group (Fig. 4c). These findings collectively show that the substantially altered lipid metabolic process in NASH was efficiently improved, and DNL was in part inhibited by the isorhamnetin treatment.

### Changes in oxidation reduction process and activation of HSCs

**Figure 5:**
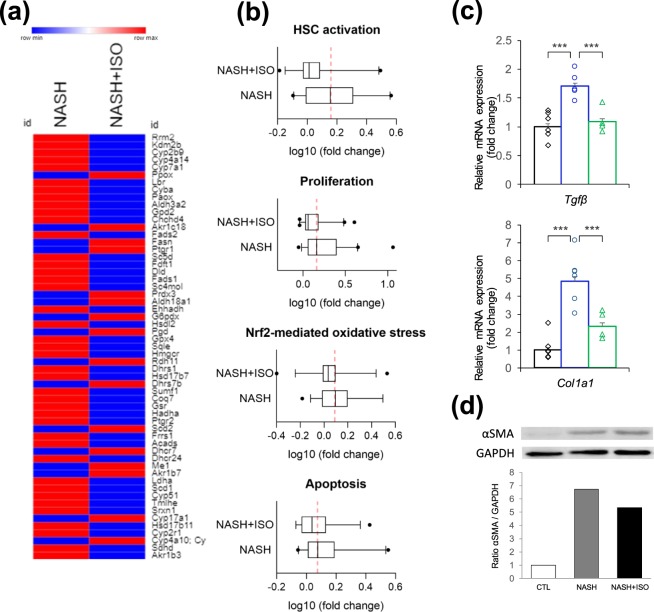
Changes in oxidation reduction process and activation of HSCs. (**a**) Heat map showing DEGs involved in oxidation reduction process between NASH and NASH + ISO compared to CTL group. (**b**) Box plot with median averaging genes involved in HSC activation (26 genes averaged), proliferation (41 genes averaged), Nrf2-mediated oxidative stress (38 genes averaged), and apoptosis (24 genes averaged). Red dash line shows the median of NASH group (**c**). Relative mRNA expression level of TGFβ and collagen type I genes (n = 4–6/group) by qPCR analysis. Data are shown as mean ± SEM with significance ***p < 0.001. (**d**) Immunoblot analysis for αSMA in liver tissue and quantification of band intensities normalized to GAPDH.

Oxidative stress is well known to be one of the main drivers in the development of fibrosis and cell death by causing cell damage and liver injury^30,31^. In response to hepatic insults and increased secretion of pro-inflammatory cytokines such as transforming growth factor beta (TGFβ), HSCs become activated from the quiescent state which leads to excessive deposition of extracellular matrix (ECM) and eventually scarring. Expression level of genes implicated in oxidation reduction process was greatly elevated in NASH (Fig. S3). When all upregulated redox gene expressions (57 genes in total) occurred in NASH were compared with NASH + ISO in heat map, important proportion of genes (41 genes) expression was decreased in NASH + ISO (Fig. 5a). To yield a clearer understanding of the effect of isorhamnetin on redox-mediated processes, we further analyzed genes implicated in HSCs activation, proliferation, Nrf2-mediated oxidative stress and apoptosis. The mean expression level of genes involved in HSC activation such as transforming growth factor beta receptor 1 (*Tgfbr1*) and several types of collagen was significantly greater in NASH, and decreased to near CTL level in NASH + ISO, indicating the activation of HSCs was decreased after isorhamnetin treatment (Fig. 5b and Supplementary Dataset). Among 41 genes averaged in proliferation process such as several genes encoding cell division cycle-associated proteins (*Cdca2, Cdca3, Cdca8*), globally upregulated genes were overrepresented in NASH, while downregulated in NASH + ISO. Nrf2-mediated oxidative stress markers including 38 genes were slightly ameliorated in NASH + ISO compared to NASH. Similarly, genes included in regulation of apoptosis encoding such as TNF-receptor superfamily proteins (*Tnf, Tnfrsf11b, Tnfrsf12a, Tnfrsf21*), Fas cell surface death receptor (*Fas*), and caspases (*Casp2, Casp6, Casp7*) were higher in NASH compared to NASH + ISO. TGFβ is known to mediate hepatic fibrosis by activating HSCs which result in increased ECM production^32^. Thus, we measured individual mRNA expression level of TGFβ, and collagen type I. The results indicated that the NASH + ISO group showed consistently decreased mRNA levels compared to NASH (p < 0.001 for both genes) (Fig. 5c). In addition, αSMA, a marker of activated HSCs, protein level markedly increased in NASH but decreased in NASH + ISO group (Fig. 5d). Our data taken together suggest that isorhamnetin treatment prevented not only HSC activation but also mitigated oxidative stress and activation of proliferation and apoptosis pathways in NASH-induced liver.

### Reduced macrophage infiltration in adipose tissue of NASH-induced mice treated with isorhamnetin

**Figure 6:**
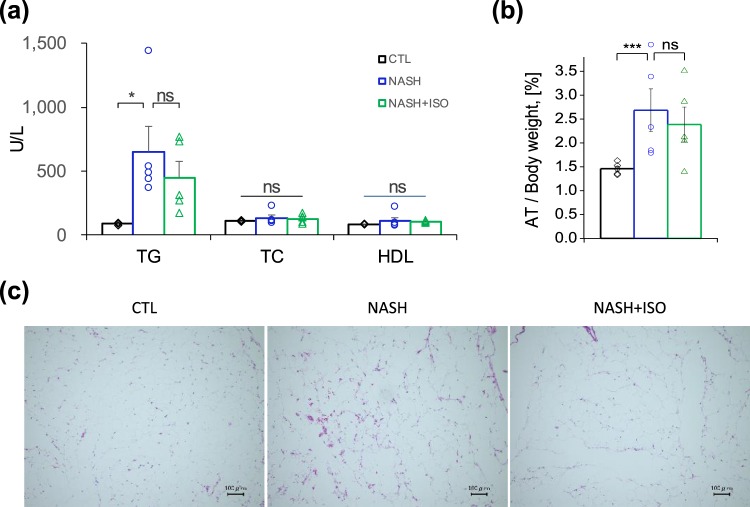
Effect of isorhamnetin on adipose tissue and lipid profile in serum. (**a**) Serum level of TG, TC, and HDL measured by automated biochemical analyzer (n = 5/group, CTL vs. NASH: p = 0.0215). (**b**) Adipose tissue on body weight ratio (n = 7/group). Data are shown as mean ± SEM with significance *p < 0.05, ***p < 0.001 and ns = non-significant. (**c**) Representative microscopic images of HE-stained adipose tissue sections (scale bar = 0.1 mm).

We next sought to understand whether the effect of isorhamnetin on steatosis amelioration observed in NASH + ISO liver was also mediated by white adipose tissue amelioration. Increased fatty acid influx derived from adipocytes lipolysis contribute up to 60% of liver fat depot^27^. We have evaluated serum lipid profile and inflammatory condition of adipose tissue using histological analysis. Serum lipid profile, including total cholesterol (TC), and high-density lipoprotein (HDL) did not show a marked difference among all groups. Serum TG level was greater both in NASH and NASH + ISO mice compared to CTL group (Fig. 6a). Adipose tissue to body weight ratio was significantly increased in NASH-induced mice than CTL group (2.72% and 1.42% for NASH and CTL, respectively), but not statistically significant from NASH + ISO although a slight decrease was observed in NASH + ISO (2.72% and 2.38% for NASH and NASH + ISO, respectively) (Fig. 6b). Most importantly, HE-stained adipose tissue showed an important accumulation of macrophage infiltration in NASH, although adipocyte hypertrophy did not display visible difference between NASH and NASH + ISO groups (Fig. 6c). Moreover, adipogenesis was effectively inhibited by the isorhamnetin supplementation in adipocytes (Supp. Fig. S4).

## Discussion

Researchers still have been seeking an effective drug although the underlying molecular mechanism responsible for etiology of NASH is not completely elucidated yet. Several studies showed curative and preventive effects of natural flavonoids against the liver diseases^20,33^. A recent rodent NASH model that we used in our study can be induced within four weeks and was able to show the set of main hallmarks present in human NASH, including liver steatosis, injury and apoptosis, and fibrosis. This advantage allowed us to study the effect of isorhamnetin not only in liver itself but also in systemic level. In this study, we demonstrated that oral administration of isorhamnetin could consistently alleviate pathologic features of NASH when compared to non-treated NASH-induced mice with the intention of finding a beneficial dietary supplement to drug development.

Liver steatosis, inflammation, and fibrosis are interrelated and are likely to aggravate each other by positive feedback leading to progression of NASH^34^. Lipid accumulation in the liver precedes the onset of NASH arising from three main sources: around 60% from fat-rich diet, 10–20% from lipolysis of adipose tissue, and 20–30% from hepatic DNL^27,35^. In current clinical practice, diet source and lipolysis of adipose tissue can be controlled up to certain extent by managing diet regime combined with pharmacological strategies. In NAFLD patients, DNL pathway in liver is constantly activated because of insulin resistance and contributes to exacerbation of hepatic steatosis^36–38^. Main regulator of DNL pathway is insulin-induced SREBP1c which triggers its downstream gene expressions required for fatty acid synthesis. SREBP1c is activated following the cleavage in Golgi and matured in endoplasmic reticulum. Overactivation of SREBP1c induces endoplasmic reticulum stress which associates with liver steatosis due to increased oxidative stress^39^. Hepatic lipid content was reduced by isorhamnetin treatment from 37% to 23% which brings NASH-induced liver back to mild steatosis category (5–33% as mild, and 34–66% as moderate) according to steatosis grade of NAS score^40^. We found that isorhamnetin treatment of NASH-induced mice could attenuate DNL pathway by regulating lipogenic key transcription factor, SREBP1c, which, in turn, downregulated lipogenic enzymes. This result correlates with the histological findings of reduced hepatic TG content and intrahepatic lipid accumulation in isorhamnetin-treated NASH mice compared to untreated NASH mice. It is seemingly possible that the inhibition of DNL pathway may be resulted in the alleviation of liver steatosis by 20–30% because the mRNA expression level of ApoB was not differed by the isorhamnetin treatment. ApoB is a protein synthesized in liver and is required for the formation of very-low-density lipoprotein (VLDL), which exports TG from liver and delivers to body tissues, including adipose tissue. Previous studies demonstrated that hepatic ApoB production is decreased in NASH patients resulting in diminished secretion of VLDL and increased hepatic steatosis^41^.

Yu Zhang *et al*. showed that four weeks of isorhamnetin treatment could reduce the body weight significantly in diet-induced NAFLD mice and leptin-deficient obese mice model^42^. Moreover, previous metabolic studies have also observed the difference in body weight among groups after at least four weeks of treatment with isorhamnetin^42,43^. Thus, in our study, two weeks of treatment was more likely premature to observe a detectable difference in body weight and liver weight between NASH and NASH + ISO although the liver weight was slightly reduced in NASH + ISO group. On the other hand, at systemic level isorhamnetin was also reported to improve glucose metabolism and insulin sensitivity, and protect against lipid peroxidation in diet-induced obese mice and streptozotocin-induced diabetic rats^44^. We found that the liver injury markers were greatly decreased following the isorhamnetin treatment.

The mechanism that underlies hepatic fibrogenesis is primarily initiated by the activation of HSCs in response to hepatic insults. TGFβ, a potent fibrogenic cytokine, plays a pivotal role in the activation of HSC by triggering its effector molecules to stimulate fibrogenic gene expressions and to induce collagen production. The ensembles of genes implicated in HSC activation, proliferation, and apoptosis were downregulated in NASH + ISO accompanied by the downregulation of genes involved in Nrf-mediated oxidative stress. In addition, mRNA expression of TGFβ and collagen by isorhamnetin were reduced suggesting that isorhamnetin may also exert its biological activity on HSCs which are the main sources of TGFβ secretion and TGFβ-mediated production of collagen in ECM. Yang *et al*. reported that isorhamnetin isolated from *oenanthe javanica* exerted anti-fibrotic effect in mice liver with CCl_4_-induced fibrosis by preventing the activation of TGFβ-induced smad2/3 pathway^19^. In our study, we did not exclude possible inflammatory insults from adipose tissue and hepatic steatosis-related intrahepatic deregulation of gene expression since the ‘second hits’ possibly act as positive feedback to exaggerate ‘first hits’. In this study, we showed that isorhamnetin could prevent the activation of TGFβ-mediated fibrogenesis in NASH-induced mice. Additionally, the release of apoptotic bodies derived from injury-induced parenchymal cell apoptosis, activation of immune cells due to systemic inflammation, signaling from Kupffer cells, and lipid peroxidation are considered as fibrogenic factors leading to HSCs activation^45^. Chronic fibrotic state and hepatic cell death by apoptosis are positively correlated with the severity of NASH^26,46^. We have found that gene expressions related to apoptosis and the number of apoptotic cells in liver were greatly reduced in the treated group. These results suggest that the isorhamnetin treatment may reverse in longer-term fibrosis and liver injury in NASH by mitigating systemic inflammation as well as by preventing HSCs activation.

Obesity, insulin resistance, and type 2 diabetes are all considered as risk factors for the development of NAFLD and NASH, which are primarily characterized by an ectopic accumulation of lipid in liver. Adipose tissue, especially visceral one, is known to be responsible for elevated lipolysis and systemic inflammation due to insulin resistance resulting in hepatic lipid accumulation and inflammation^34^. Although the lipid profile measured in non-fasting serum demonstrated insignificant difference between treated and non-treated NASH-induced mice, adipose tissue of NASH-induced mice was more inflamed, as evidenced by the increased number of macrophage infiltration, while adipocytes of NASH + ISO were greatly ameliorated. Of note, similar studies that used flavonoids to treat HFD-induced metabolic disorders in rodents also noted indifference of lipid profile in serum^21^ but found the net amelioration of systemic inflammation accompanied with reduced adipose tissue and body weight after longer duration of treatment in diet-induced mice or *db/db* mice^18,42^.

Altogether, our results demonstrated that isorhamnetin elicits a beneficial effect on hallmarks of NASH by improving steatosis, injury, and fibrosis in a human-like NASH-induced mouse model. This hepatoprotective effect of isorhamnetin was correlated to the inhibition of DNL and fibrogenic gene expressions; the alleviation of liver TG content; and the diminution of hepatic collagen deposition accompanied with the reduced number of apoptotic hepatocytes. Thus, isorhamnetin can be a novel candidate for the consideration of additional compound in NASH drug development.

## Material and Methods

### Animals

Six-week-old male C57BL/6J mice (Charles River Laboratories JAPAN Inc., Kanagawa, Japan) were maintained at room temperature in a 12 h light/dark cycle. After one week of acclimatization with standard chow diet and tap water *ad libitum*, all animals were randomly assigned (Day 0) into three experimental groups (n = 8/group): Control (CTL), NASH, and NASH treated with isorhamnetin (NASH + ISO). All animal procedures were approved by the Animal Study Committee of University of Tsukuba (No.17–312) and were handled according to the Guiding Principles for the Care and Use of Animals in the Field of Physiological Sciences approved by The Physiological Society of Japan.

### Chemicals

Isorhamnetin was synthesized from commercially available quercetin (Fujifilm Wako Pure Chemical Corp., Tokyo, Japan) according to the protocol^47^ and used for *in vivo* experiment. Commercially available selective agonist for LXRα and LXRβ (T0901317) (Cayman Chemical, Ann Arbor, MI, USA); and carbon tetrachloride (CCl_4_) (Wako Pure Chemical Industries Ltd., Osaka, Japan) were used.

### Experimental design and procedure

NASH was induced according to the protocol^8^. Briefly, the CTL group mice were fed with laboratory chow diet and received vehicles. NASH and NASH + ISO groups were subjected to high fat diet (60% cal from fat, D12492, Research Diets Inc., New Brunswick, NJ, USA) from Day 0–24. Rodents received intraperitoneal injections of CCl_4_ at 0.1 ml/kg of body weight 4 times (Day 14, 17, 21, and 24), and T0901317 at 2.5 ml/kg of body weight 5 times (Day 20–24) to induce NASH. Corn oil and dimethyl sulfoxide (DMSO) were used as a vehicle for CCl_4_ and T0901317 injections respectively. NASH + ISO group was treated with daily oral administration of isorhamnetin at 50 mg/kg of body weight (vehicle for CTL and NASH group) for last two weeks (Day 11–24). Body weight and food intake were measured every day. At the end of experiment (Day 25) blood was collected from retro-orbital sinus using capillary tube after mice are slightly anesthetized with isoflurane inhalation. Serum was separated by centrifugation at 3000 rpm for 10 min and was stored at −20 °C until biochemical analysis. Liver and epididymal fat were isolated immediately after the exsanguination. Median lobe of liver was stocked in either liquid nitrogen or cryopreserves for further analysis. The remaining liver was fixed in 10% neutral buffered formalin for histological analysis.

### Blood biochemical analysis

The TC, TG, HDL, ALT, and AST levels in serum were measured using FUJI DRI-CHEM 7000 automated chemistry analyzer (Fujifilm Corp. Tokyo, Japan).

### Histological analysis

Tissues embedded in paraffin were sliced into sections and stained with H&E, and Sirius red using the standard protocol for histopathology analysis of liver. Cryopreserved liver tissues were sliced and used for oil red O staining with hematoxylin counterstaining. Stained slides were observed under a BZ-9000 BioRevo digital microscope (Keyence Corp., Osaka, Japan), and were analyzed using ImageJ.

### Apoptosis TUNEL assay

Apoptotic cells *in situ* were detected in paraffin-embedded liver sections using DeadEnd Colorimetric TUNEL System (Promega Corp., Madison, WI, USA) according to the manufacturer’s instruction. Sections were counter stained with hematoxylin. Stained slides were observed under a BZ-9000 BioRevo digital microscope (Keyence Corp., Osaka, Japan), and were analyzed using ImageJ.

### Gene expression analysis

The total RNA of liver tissues was extracted using ISOGEN reagent (Nippon Gene Co., Ltd. Toyama, Japan) according to the manufacturer’s protocol. First-strand cDNA was amplified from the total RNA (100 ng) using SuperScript III Reverse Transcriptase kit (Invitrogen, Carlsbad, CA, USA). The quantification of total RNA and cDNA was measured on NanoDrop 2000 spectrophotometer (Thermo Scientific, Wilmington, DE, USA). Real-time quantitative PCR of target gene expression was assayed by TaqMan predesigned primers (Applied Biosystems, Foster City, CA, USA) and TaqMan Gene Expression Master Mix (Life Technologies, Carlsbad, USA) using the 7500 Fast Real-Time PCR System (Applied Biosystems, Foster City, CA, USA). The following primers were bought from Applied Biosystems: sterol regulatory element binding protein 1 (*Srebf1*) (Mm00550338_m1), fatty acid synthase (*Fasn*) (Mm00662319_m1), acetyl-Coenzyme A carboxylase alpha (*Acaca*) (Mm01304257_m1), transforming growth factor beta 1 (*Tgfb1*) (Mm01178820_m1), collagen type 1 alpha 1 (*Col1a1*) (Mm00801666_g1), apolipoprotein B (*Apob*) (Mm01545150_m1), and glyceraldehyde-3-phosphate dehydrogenase (*Gapdh*) (Mm99999915_g1). The 2^−ΔΔCt^ method was applied to calculate the relative mRNA expression levels using *Gapdh* as a housekeeping endogenous control.

### Total RNA extraction and microarray analysis of liver

The total RNA of liver tissues was extracted using ISOGEN reagent (Nippon Gene Co., Ltd. Toyama, Japan) according to the manufacturer’s protocol. The quality and quantity of total RNA was measured on NanoDrop 2000 spectrophotometer (Thermo Scientific, Wilmington, DE, USA), and 100 ng of RNA input was used for microarray analysis. The gene expression profiling was performed using GeneChip 3′ IVT PLUS Reagent Kit (Affymetrix Inc., Santa Clara, CA, USA) with Affymetrix® 3′ IVT Array Strips for GeneAtlas® System (GeneChip® MG-430 PM) according to the manufacturer’s user guide. Raw intensity values were obtained using GeneAtlas™ Imaging Station and were normalized using Expression Console Software provided by the Affymetrix following robust multichip average (RMA) algorithm (http://www.affymetrix.com). Subsequently, the raw data set was transferred to the Transcriptome Analysis Console (TAC) v4.0 (ThermoFisher inc.). A threshold with fold-change ≥ 2.0 and p < 0.05 compared to CTL were considered as DEGs. Further analyses were conducted using Functional annotation tool of The Database for Annotation, Visualization and Integrated Discovery (DAVID) v6.8 online bioinformatics database to identify enriched Gene Ontology (GO) and KEGG pathways. Transcriptome dataset is available at NCBI Gene Expression Omnibus database (www.ncbi.nlm.nih.gov/geo) with accession number GSE137635. Heat maps were visualized with DEGs in each biological process identified by GO using Morpheus online tool (https://software.broadinstitute.org/morpheus). Box plots analyses were visualized using GraphPad Prism 8 which show the average of genes implicated in each process (grouping of genes by biological process as mentioned in Cazanave *et al*.^48^).

### Western blotting

Total protein was extracted from liver tissue (50 mg) using radio immunoprecipitation assay (RIPA) buffer (Sigma-Aldrich, St. Louis, USA) containing protease inhibitor cocktail (P8340, Sigma-Aldrich, St. Louis, MO, USA), and quantified with 2-D Quant kit (GE Healthcare, Chicago, USA) following the manufacturer’s instruction. Protein samples (20 μg) were resolved in 10% SDS-PAGE and transferred to nitrocellulose membrane. After blocking in Odyssey blocking buffer (LI-COR, NE, USA) for 2 hours, membranes were blotted with primary antibody for overnight at 4 °C and incubated with appropriate Alexa Fluor® conjugated secondary antibodies. Imaging and quantification of signal intensity were detected using the Odyssey Fc Imaging System (LI-COR, NE, USA). Primary antibodies: rabbit anti-αSMA (ab5694), rabbit anti-FAS (ab22759), rabbit anti-SREBP1 (ab28481), mouse anti-GAPDH (ab8245), and secondary antibodies: Alexa Fluor® 488-conjugated donkey anti-rabbit (ab150073), Alexa Fluor® 594-conjugated donkey anti-mouse (ab150108) were purchased from Abcam. Uncropped images of western blotting gel are available in the Supplementary Files.

### Quantification of liver triglyceride

Total TG content in the liver was quantified using commercially available colorimetric kit (Cayman Chemical, Ann Arbor, MI, USA).

### Statistical analysis

All data are expressed as the mean ± SEM, unless otherwise mentioned. Normality test was performed to confirm the normal distribution. A one-way analysis of variance (ANOVA) followed by Tukey’s posthoc test was applied to assess the statistical significance of difference among the treatment groups. Statistical outliers were excluded from data analysis. A value of p < 0.05 was considered significant for all results. Statistical analyses were performed using IBM SPSS Statistics version 24.0.

## Supplementary information

Supplementary information

Supplementary Dataset 1

## Data Availability

All data generated or analyzed during this study are included in this published article and its Supplementary Files. Microarray data are deposited in the Gene Expression Omnibus (GEO) under Accession Number: GSE137635.
