# Supplementary material for: Isorhamnetin Alleviates Steatosis and Fibrosis in Mice with Nonalcoholic Steatohepatitis
Source: Sci Rep. 2019 Nov 7;9:16210. doi: 10.1038/s41598-019-52736-y (PMC6838085; doi:10.1038/s41598-019-52736-y)
Supplement: Supplementary file 1 — Supplementary information [file 41598_2019_52736_MOESM1_ESM.pdf]

Isorhamnetin Alleviates Steatosis and Fibrosis in Mice with Nonalcoholic Steatohepatitis

Munkhzul Ganbold[1], Yohei Owada[2], Yusuke Ozawa[2], Yasuhiro Shimamoto[3], Farhana Ferdousi[4], Kenichi Tominaga[3], Yun-Wen Zheng[2], Nobuhiro Ohkohchi[2], Hiroko Isoda[1,3,4,5,*]

[1] School of Integrative and Global Majors (SIGMA), University of Tsukuba, Tsukuba, Ibaraki 305-8572, Japan

[2] Department of Gastrointestinal and Hepato-Biliary-Pancreatic Surgery, Faculty of Medicine, University of Tsukuba, Tsukuba, Ibaraki 305-8575, Japan

[3] Interdisciplinary Research Center for Catalytic Chemistry, National Institute of Advanced Industrial Science and Technology (AIST), Tsukuba, Ibaraki 305-8565, Japan

[4] Alliance for Research on the Mediterranean and North Africa (ARENA), University of Tsukuba, Tsukuba, Ibaraki 305-8577, Japan

[5] Faculty of Life and Environment Science, University of Tsukuba, Tsukuba, 305-8572, Japan

*Corresponding author. isoda.hiroko.ga@u.tsukuba.ac.jp, Faculty of Life and Environmental Sciences, University of Tsukuba, 1-1-1 Tennodai, Tsukuba, Ibaraki 305-8572, Japan. Tel: +81 29 853 5775; Fax: +81 29 853 5776.

Contents

Supplementary methods 3

Histological analysis 3

Cell lines and culture condition 3

Cell viability assay 3

Lipid accumulation in HepG2 cells 3

Lipogenesis in 3T3-L1 cells 4

Supplementary figures 5

Supplementary figure S1. 5

Supplementary figure S2. 6

Supplementary figure S3. 7

Supplementary figure S4. 8

Figure 4d. 9

Figure 4d. 10

Figure 5d. 11

Supplementary methods

Histological analysis

Liver tissues embedded in paraffin were sliced into sections and stained with Masson's trichrome using standard protocol for histopathology analysis. Stained slides were observed under a BZ-9000 BioRevo digital microscope (Keyence Corp., Osaka, Japan).

Cell lines and culture condition

Human hepatocyte carcinoma cell line (HepG2) from Riken BRC Cell Bank (Tsukuba, Japan), rat hepatic stellate cell line (HSC-T6) from Millipore (EMD Millipore Corp., CA, USA), and mouse preadipocyte cell line (3T3-L1) from JCRB Cell Bank (Ibaraki, Japan) were obtained respectively. HepG2 and 3T3-L1 cells were maintained in Dulbecco's modified Eagle's medium (DMEM) (D6046, Sigma-Aldrich, Japan), and HSC-T6 cells were maintained in DMEM (D5796, Sigma-Aldrich, USA) supplemented with 10% fetal bovine serum (FBS) (Gibco, USA) and 1% penicillin (5000 μg/ml) – streptomycin (5000 IU/ml) (Lonza, Japan) at 37°C in 5% CO2 humidified incubator. Cells were subcultured after reaching 80% confluence. Cells between passage number 3-9 were used for experiments.

Cell viability assay

HepG2 and HSC-T6 were maintained according to the manufacturer's instruction. Cells were seeded at the density of 1x10^5 live cells / ml in 96-well plate. After overnight incubation, cells were treated with the various doses of isorhamnetin for 24 h before adding 3-(4,5-dimethylthiazol-2-yl)-2,5-diphenyltetrazolium bromide (MTT) solution. The formed formazan crystals were dissolved in 10% sodium dodecyl sulfate (SDS) solution and absorbance was measured at 570 nm. Cell viability was calculated as Cell viability (%) = [(mean OD value of treated wells – mean OD value of the blanks) ×100%] / (mean OD value of untreated wells – mean OD value of the blanks).

Lipid accumulation in HepG2 cells

HepG2 cells were seeded at the density of 1x10^5 live cells / ml in 6-well plate, and after the overnight incubation the cells were subjected to serum free medium for 24 h of starvation. Then, the cells were incubated with 0.5 mM of oleic (OA) and palmitic acid (PA) (molar ratio of OA:PA = 2:1) to induce lipid accumulation for 24 h in the presence or absence of isorhamnetin. Accumulated lipids were stained with oil red O and dissolved in isopropanol. Absorbance was measured at 510 nm, and the lipid content was calculated as Lipid content

(%) = [(mean OD value of treated wells – mean OD value of the blanks) ×100%] / (mean OD value of untreated wells – mean OD value of the blanks).

Lipogenesis in 3T3-L1 cells

3T3-L1 cells were maintained according to the manufacturer's instruction. Adipogenesis assay kit (Cayman Chemical, MI, USA) was used to induce adipogenesis according to the manufacturer's protocol. Briefly, cells were seeded at the density of 1x10^5 live cells / ml in 6-well plate. Two days after the confluence day (Day 0), the medium was changed to induction medium containing 500 μM 3-isobutyl-1-methylxantine (IBMX), 10 μg/ml insulin, and 1 μM dexamethasone. From Day 3 maintenance medium containing 10 μg/ml insulin was changed every other day in the presence or absence of isorhamnetin until the Day 14. Formation of lipid droplets were stained with oil red O and counterstained with hematoxylin for observation.

Supplementary figures

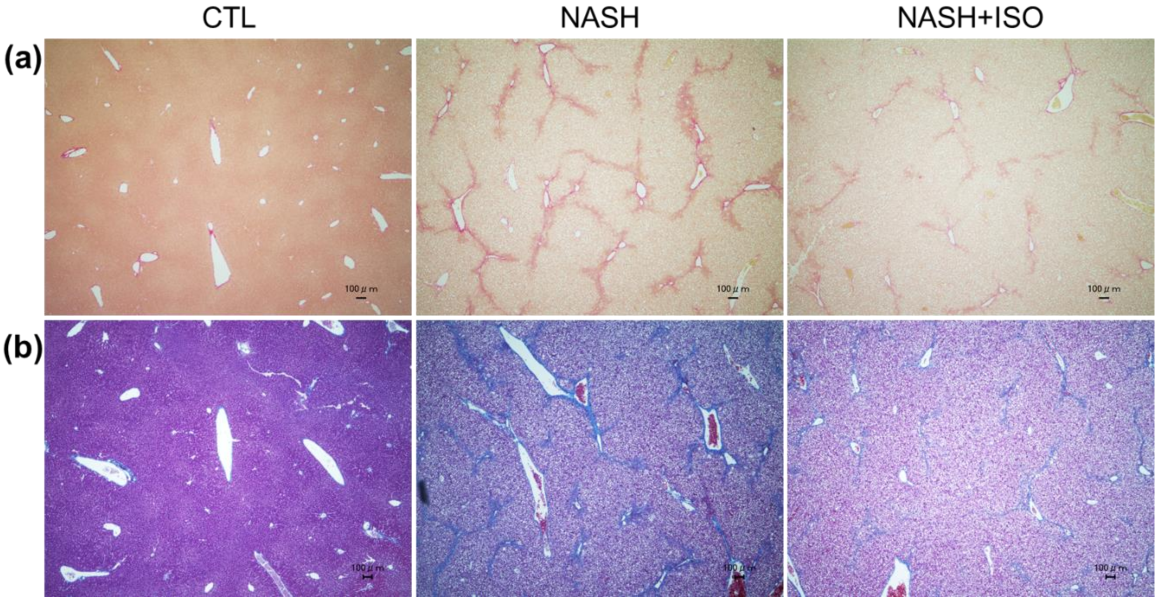

Supplementary figure S1. Representative microscopic images of Sirius red (a) and Masson's trichrome (b) -stained liver sections showing fibrosis (Scale bar = 0.1 mm).

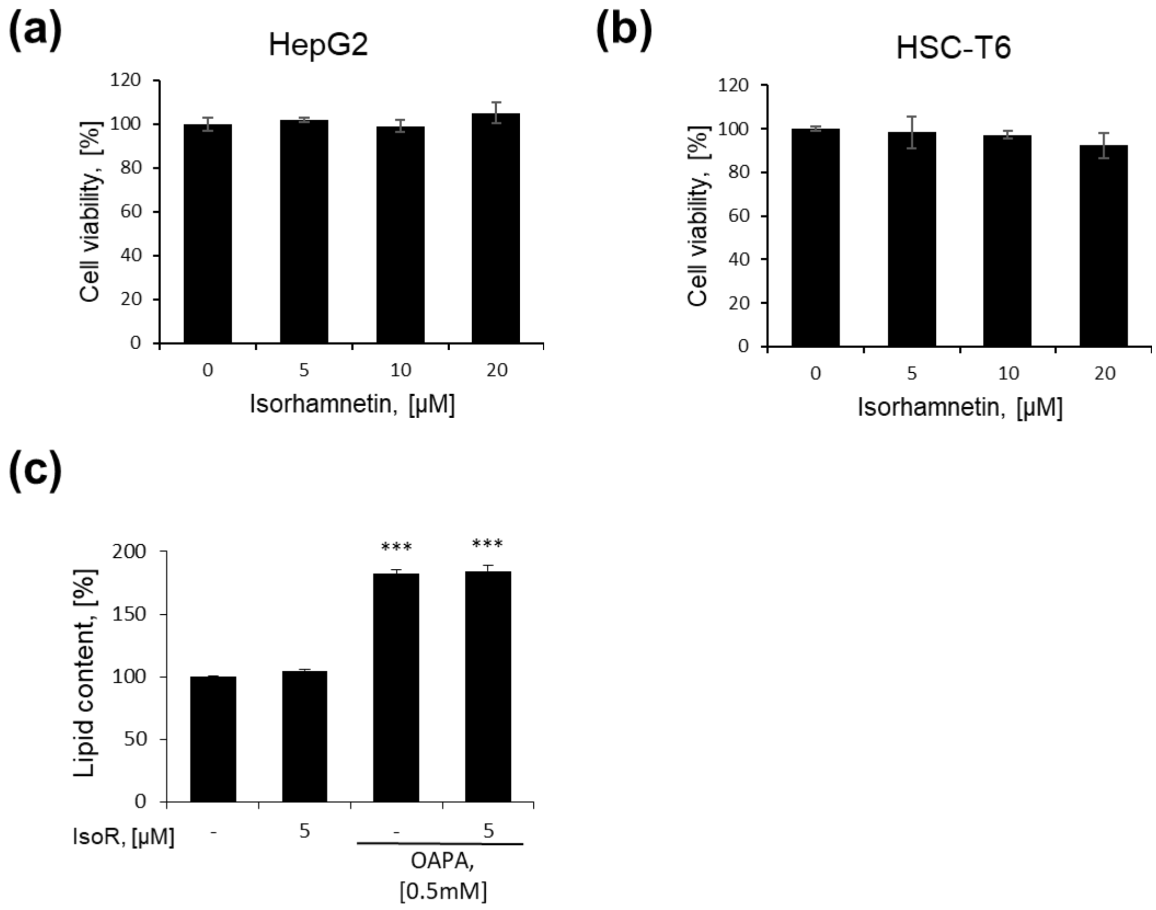

Supplementary figure S2. Effect of isorhamnetin on HepG2 and HSC-T6 cells. Cell viability of HepG2 cells (a) and hepatic stellate cells (HSC-T6) (b) treated with different concentration of isorhamnetin for 24 h measured by MTT assay. (c) HepG2 cells were starved in serum free medium for 24 h, followed by the isorhamnetin treatment in the absence and presence of the mixture of oleic acid and palmitic acid (OAPA) at 0.5mM for 24 h. Lipid content was measured by the absorbance of oil red O at 510nm. Data are shown as mean ± SD with significance *** $p < 0.001$.

Term	Count	PValue
GO:0006629~lipid metabolic process	58	4.73E-15
GO:0055114~oxidation-reduction process	57	5.76E-08
GO:0043066~negative regulation of apoptotic pr	41	1.68E-04
GO:0006974~cellular response to DNA damage st	40	3.72E-07
GO:0008152~metabolic process	40	4.16E-06
GO:0051301~cell division	35	3.35E-06
GO:0008284~positive regulation of cell proliferat	34	0.006467
GO:0006281~DNA repair	31	6.05E-06
GO:0006631~fatty acid metabolic process	27	2.66E-10
GO:0008203~cholesterol metabolic process	19	6.48E-09

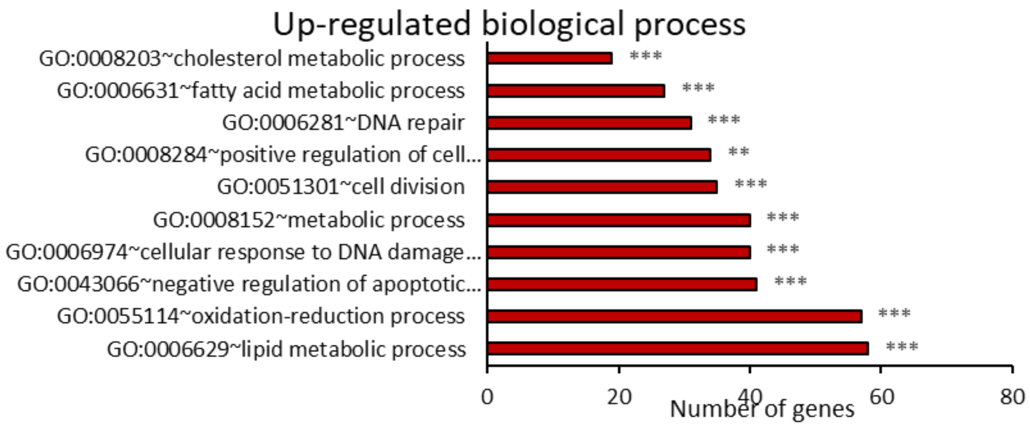

Supplementary figure S3. Gene ontology of up-regulated genes in NASH compared to CTL

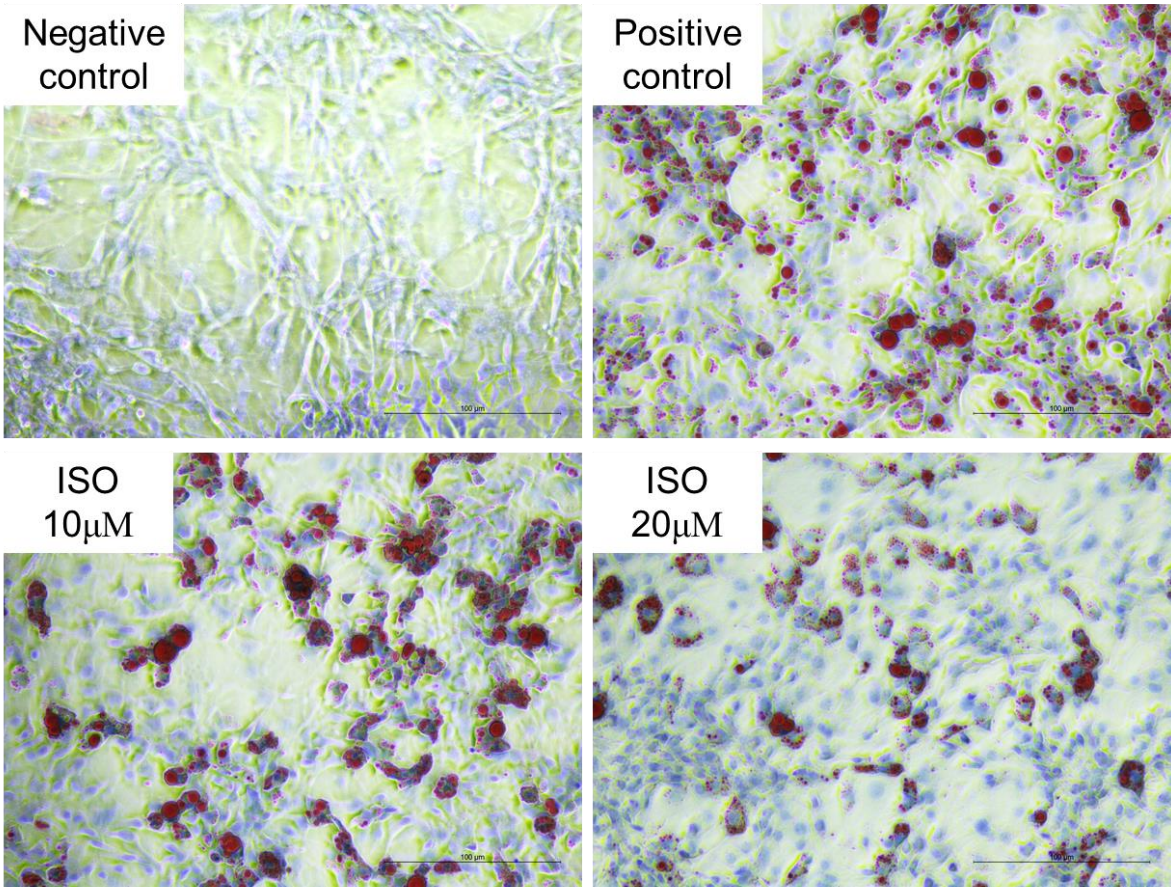

Supplementary figure S4. Effect of isorhamnetin on lipogenesis in 3T3-L1 cells. Adipocyte differentiation were induced (or not induced in Negative control) and maintained for 14 days in the absence (Positive control) and presence of different concentration of isorhamnetin. Lipid droplets were visualized with oil red O counterstained with hematoxylin (Scale bar = 0.1 mm).

(a)

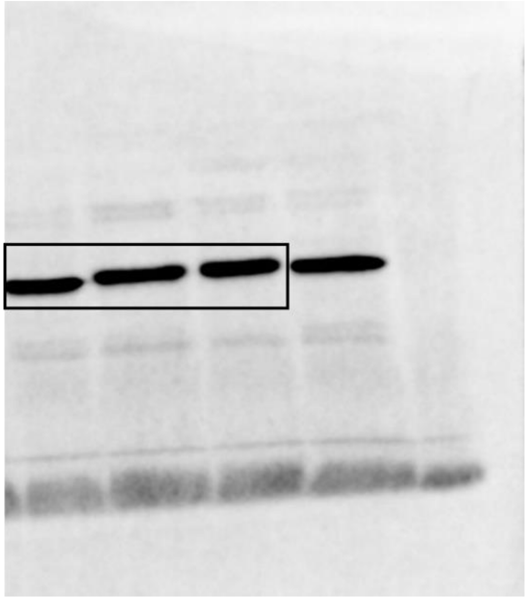

(b)

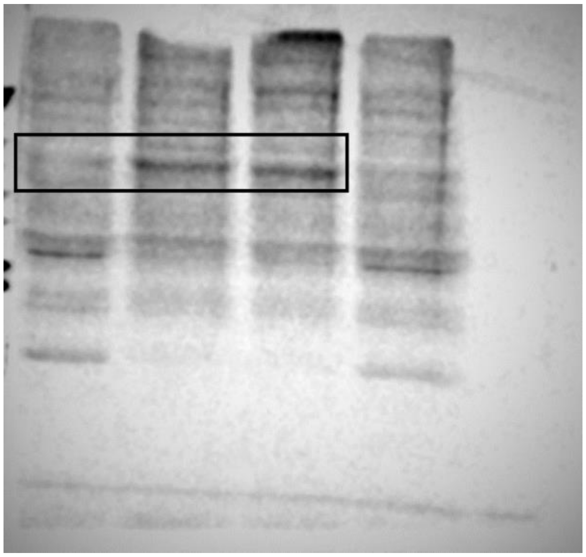

Figure 4d. (a) GAPDH, (b) SREBP1c

(a) (b)

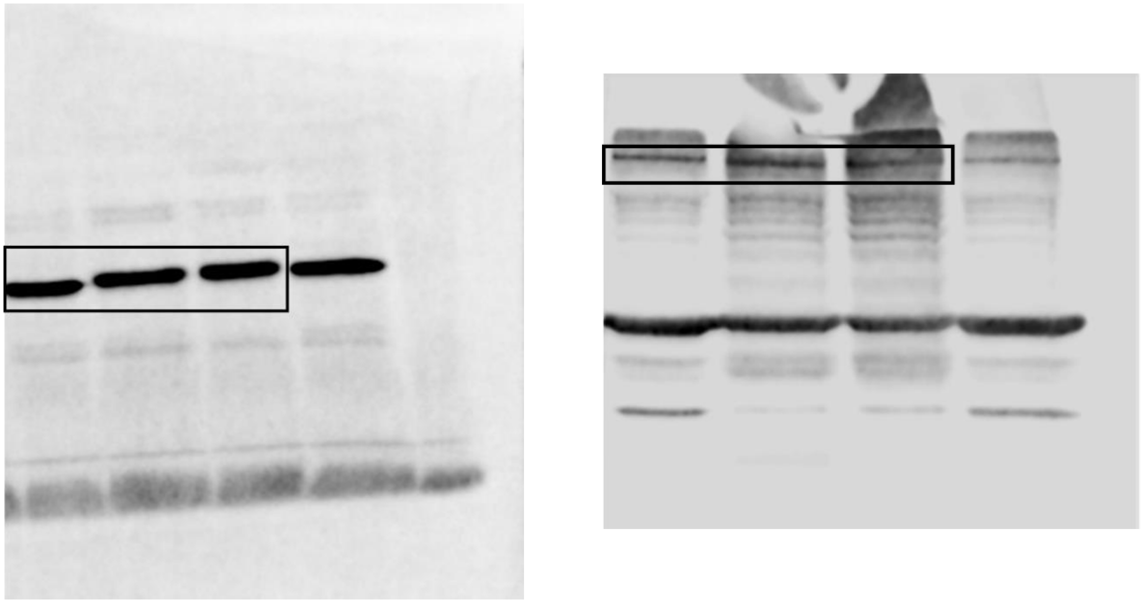

Figure 4d. (a) GAPDH, (b) FAS

(a) (b)

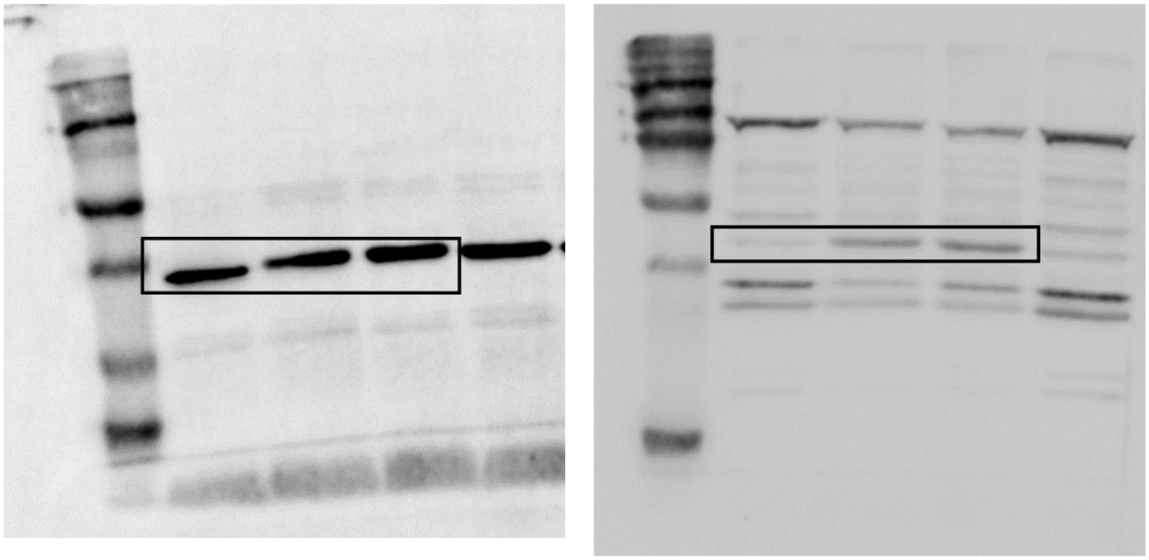

Figure 5d. (a) GAPDH, (b) aSMA
